# The Reduction in Medical Errors on Implementing an Intensive Care Information System in a Setting Where a Hospital Electronic Medical Record System is Already in Use: Retrospective Analysis

**DOI:** 10.2196/39782

**Published:** 2022-08-31

**Authors:** Yusuke Seino, Nobuo Sato, Masafumi Idei, Takeshi Nomura

**Affiliations:** 1 Department of Intensive Care Medicine Tokyo Women's Medical University Tokyo Japan; 2 Department of Anesthesiology and Intensive Care Medicine Yokohama City University Yokohama Japan

**Keywords:** clinical information system, electronic medical record, intensive care unit, medical error

## Abstract

**Background:**

Although the various advantages of clinical information systems in intensive care units (ICUs), such as intensive care information systems (ICISs), have been reported, their role in preventing medical errors remains unclear.

**Objective:**

This study aimed to investigate the changes in the incidence and type of errors in the ICU before and after ICIS implementation in a setting where a hospital electronic medical record system is already in use.

**Methods:**

An ICIS was introduced to the general ICU of a university hospital. After a step-by-step implementation lasting 3 months, the ICIS was used for all patients starting from April 2019. We performed a retrospective analysis of the errors in the ICU during the 6-month period before and after ICIS implementation by using data from an incident reporting system, and the number, incidence rate, type, and patient outcome level of errors were determined.

**Results:**

From April 2018 to September 2018, 755 patients were admitted to the ICU, and 719 patients were admitted from April 2019 to September 2019. The number of errors was 153 in the 2018 study period and 71 in the 2019 study period. The error incidence rates in 2018 and 2019 were 54.1 (95% CI 45.9-63.4) and 27.3 (95% CI 21.3-34.4) events per 1000 patient-days, respectively (*P*<.001). During both periods, there were no significant changes in the composition of the types of errors (*P*=.16), and the most common type of error was medication error.

**Conclusions:**

ICIS implementation was temporally associated with a 50% reduction in the number and incidence rate of errors in the ICU. Although the most common type of error was medication error in both study periods, ICIS implementation significantly reduced the number and incidence rate of medication errors.

**Trial Registration:**

University Hospital Medical Information Network Clinical Trials Registry UMIN000041471; https://center6.umin.ac.jp/cgi-open-bin/ctr_e/ctr_view.cgi?recptno=R000047345

## Introduction

### Background

Clinical information systems in intensive care units (ICUs), such as intensive care information systems (ICISs), have been developed to aggregate patient information, improve operational efficiency, and obtain accurate records. A commercial ICIS consists of a critical care flowsheet; computerized physician order entry (CPOE); and interfaces with bedside monitors, ventilators, and other external devices. It also has the capability to interface with other hospital systems [1].

Studies have reported that ICIS implementation is associated with both desirable and undesirable effects. The desirable effects of ICISs include improved efficiency and quality of care, improved data utilization and security, and reduced documentation time [2-5]. By contrast, the undesirable effects of ICISs include the occurrences of ICIS-related errors, reduced speed and efficiency due to poor system usability, interruption of established workflows, and the risk of system failure [5-8]. Meanwhile, the effect on the length of stay in the ICU is controversial [9,10].

In particular, when both an ICIS and a hospital electronic medical record (EMR) system are used simultaneously, the differences in performance and operability of both systems, as well as the low level of interactivity between them, can lead to new errors. ICISs are generally interfaced with EMRs because EMR systems are used for many hospital tasks; on the other hand, limitations in the level and direction of information coordination can prevent the sufficient integration of EMRs and ICISs. However, if ICISs are built into EMRs as modules, the integration of both systems would improve.

### Motivation for ICIS Implementation in Our Hospital

The EMR has been used throughout Tokyo Women's Medical University Hospital since 2014. Given that the EMR was not well suited for use in the ICU, the vital sign and prescription dashboards remained separate; therefore, paper-based orders and flowsheets were used concurrently. Subsequently, a critical incident occurred in the ICU, and inadequate records became a serious issue during the investigation of the incident. As a result, the order and charting procedures in the ICU were revised for the EMR to be used more; however, as mentioned earlier, this led to an increase in staff workload. Thus, the introduction of a commercial ICIS was planned during the reorganization of ICUs at the hospital.

### ICIS Implementation and Medical Errors in the ICU

No study has focused on the changes in error incidence in ICUs after the implementation of a commercial ICIS adding to an EMR. However, some studies have reported the effects of ICIS implementation on medication errors. A comparison of a paper-based ICU and a computerized ICU with an ICIS for medication errors showed that the incidence of medical prescription errors was 3.42% (44 errors in 1286 prescriptions) in the ICU with an ICIS compared with 27.04% (331 errors in 1224 prescriptions) in the paper-based ICU [11]. By contrast, a study in a pediatric ICU reported that ICIS implementation did not significantly reduce the prescription error rate, from 8.8% (14 errors in 159 prescriptions; 95% CI 4.4-13.2) before ICIS implementation to 4.6% (12 errors in 257 prescriptions; 95% CI 2.0-7.2) 6 months after ICIS implementation [12]. A study comparing handwritten orders with CPOE orders in a cardiac ICU reported that the error rate of prescription errors decreased from 44.8% (819 errors in 1829 prescriptions) with handwritten orders to 0.8% (16 errors in 2094 prescriptions) with CPOE [13]. Similarly, there have been reports that CPOE implementation contributed to a decrease in prescription errors in an ICU and a decrease in parenteral nutrition medication errors in a neonatal ICU [14,15].

### Objectives

Although the various advantages of ICIS implementation in ICUs have been reported, the role of an ICIS in preventing errors in an ICU remains unclear. This study aimed to investigate the changes in the incidence and type of errors in the ICU before and after ICIS implementation in a setting where an EMR system is already being used and where an ICIS is not integrated with the EMR system.

## Methods

### Study Design and Setting

This study was a retrospective analysis of the errors in the general ICU (18 beds, 1:2 nurse to patient ratio) of a university hospital (1335 beds) before and after ICIS implementation by using data from an incident reporting system. An ICIS (PrimeGaia PRM-7400, Nihon Kohden Corp) was implemented in the ICU. After a step-by-step implementation lasting 3 months, the ICIS was used in all patients starting from April 1, 2019.

### Ethics Approval

The study was approved by the Institutional Review Board of Tokyo Women’s Medical University (approval #5224; June 20, 2019), and the need for informed consent was waived due to the retrospective study design. All methods in the study were performed in accordance with the relevant guidelines and regulations.

### Before ICIS Implementation (April 2018 to September 2018)

**Figure 1:**
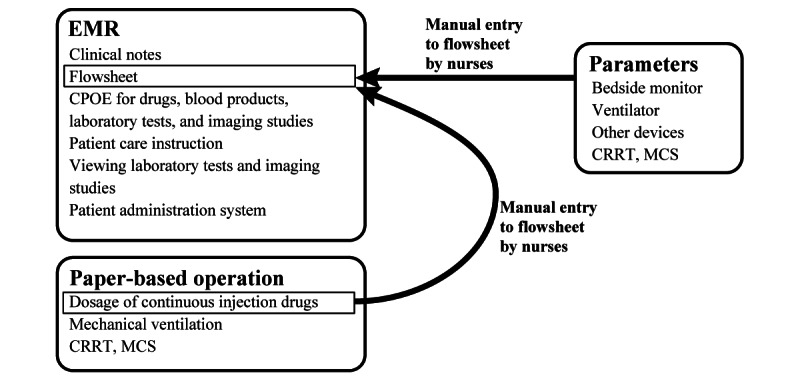
Workflow in the study period before ICIS implementation (April 1, 2018, to September 30, 2018). CPOE: computerized physician order entry; CRRT: continuous renal replacement therapy; EMR: electronic medical record; ICIS: intensive care information system; MCS: mechanical circulatory support.

An EMR system (HOPE EGMAIN, Fujitsu Japan Limited) was already in use in the ICU and has many components, including CPOE with a clinical decision support system (CDSS), documentation, flowsheet, patient care instruction, and ordering and viewing functions for laboratory tests and imaging studies. However, given that the CPOE was not optimized for use in the ICU, paper-based orders were used for the dosage of continuous injection drugs. The orders for mechanical ventilation, mechanical circulatory support, and renal replacement therapy settings were also paper based. In addition, nurses had to manually enter the dosages of continuous injection drugs; the fluid balance; and the parameters derived from bedside monitors, ventilators, and other monitors into the EMR flowsheet (Figure 1). This input process was time-consuming and contributed to the heavy workload of ICU nurses. The EMR flowsheet was not optimized as an information tool for critically ill patients and was slow to operate.

### ICIS Implementation Process

A multidisciplinary implementation project team consisting of physicians, nurses, pharmacists, clinical engineers, and hospital system engineers was formed to determine the system specifications and prepare for implementation. The development of the ICIS began in October 2017. The ICIS was rolled out in October 2018, and training sessions for physicians and nurses also began in October 2018. The ICIS was launched on January 8, 2019. Considering the smooth adaptation and heterogeneity of patients, physicians, and nurses, incremental implementation was chosen. The project team modified the system and operational procedures during implementation.

### After ICIS Implementation (April 2019 to September 2019)

**Figure 2:**
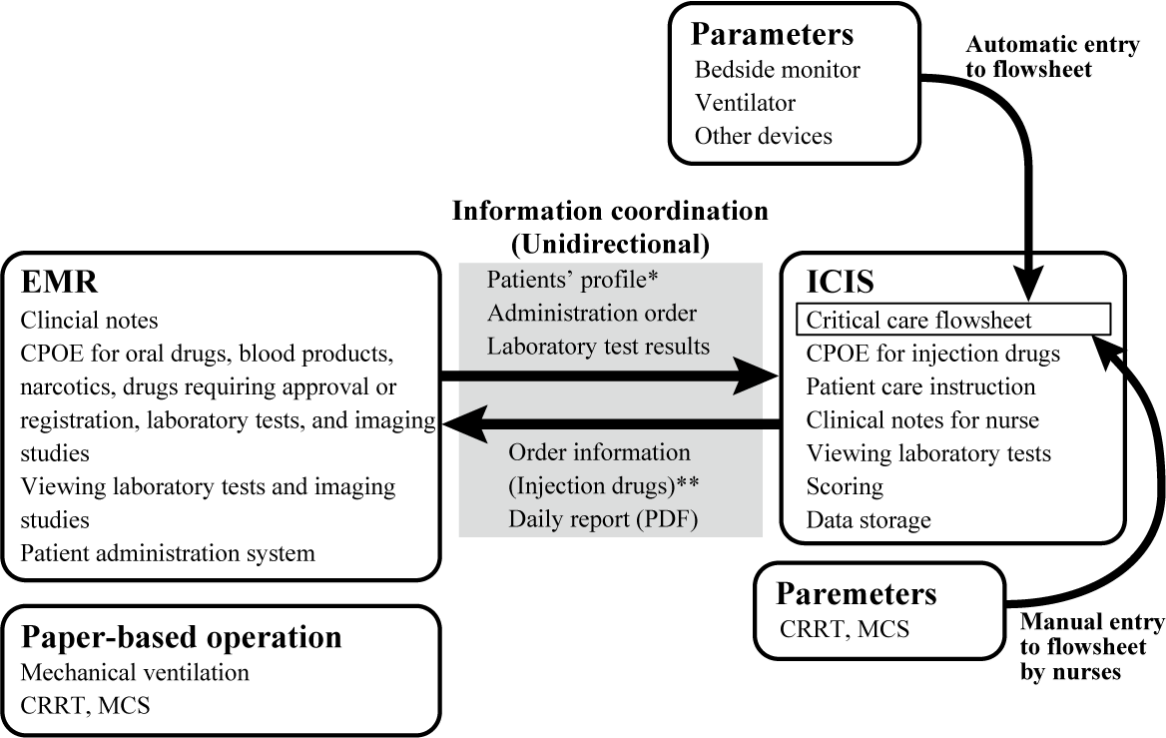
Workflow in the study period after the completion of a step-by-step implementation of ICIS (April 1, 2019, to September 30, 2019). CPOE: computerized physician order entry; CRRT: continuous renal replacement therapy; EMR: electronic medical record; ICIS: intensive care information system; MCS: mechanical circulatory support. *The patients’ basic profiles are sent from the EMR to the ICIS, except for information on their allergies and contraindications. **Blood products, narcotics, and drugs that require approval or registration (broad-spectrum antibiotics, drugs for chemotherapy, and rarely used drugs) need to be ordered in both the EMR system and ICIS. Changes in the orders are not synchronized.

The major components of the implemented ICIS included a critical care flowsheet; CPOE without CDSS; an interface with bedside physiologic monitors, ventilator, and other external devices; and an interface with an EMR system. The ICIS replaced the CPOE, flowsheet, and patient care instruction of the EMR system, and nurses no longer had to manually enter the dosages of drugs and parameters because the parameters were automatically registered into the system. However, the level of coordination between the EMR system and ICIS was low (Figure 2). Most of the drugs administered in the ICU were prescribed with the ICIS, and the ordering information was sent to the EMR system and the logistics system of the pharmacy department. In contrast, narcotics, drugs that require approval or registration (broad-spectrum antibiotics, drugs for chemotherapy, and rarely used drugs), and blood products had to be prescribed in both systems. Oral medications, laboratory tests, and imaging tests had to be ordered using the EMR system. The laboratory test results were displayed in the ICIS, while the imaging tests and their findings could be viewed only in the EMR system. The order of settings of mechanical ventilation, mechanical circulatory support, and renal replacement therapy was maintained using a paper-based system to avoid excessive workflow changes for ICU physicians and nurses. Given that the EMR system and ICIS were not integrated and that the ICU staff needed to operate both systems simultaneously, dual displays were equipped on the bedside computers to ensure operational efficiency.

### Data Collection and Outcomes

Data of ICU errors in a 6-month period 1 year before the ICIS implementation (April 1, 2018, to September 30, 2018) and 3 months after ICIS implementation (April 1, 2019, to September 30, 2019) were extracted from the incident reporting system to determine the number and incidence rate of errors. The incident reporting system in the hospital was based on voluntary self-reporting. All error reports were submitted using a computer-based form and were reviewed by safety managers in departments that handle errors and by the Patient Safety Management section. Information regarding the length of stay and patients' treatment departments in the ICU was collected from the ICIS and EMR system during the study period. We defined all events reported in the incident reporting system as errors in this study. The errors were classified into 7 types on the basis of the classification system of the Japan Council for Quality Health Care (Table 1) [16]. The errors were classified into 8 levels according to severity and influence based on the National University Hospital Council of Japan’s classification system in the incident reporting system (Table 2) [17]. The type and level of errors were preliminarily determined by the staff filling the report and were reviewed and adjudicated by safety managers in the departments that handle errors.

The primary outcomes in this study were the number and incidence rate of errors during the 6-month study period. The secondary outcomes included the number and incidence rates of errors by category and type, the patient outcome level of errors, the number and incidence rate of ICIS-related errors, and the composition of treatment departments.

**Table 1:** Classification of the type of errors recommended by the Japan Council for Quality Health Care.

Type of errors	Description
Medication	Errors related to medication or blood transfusion
Line, tube, or drain	Errors related to lines (venous routes or catheters), tubes (endotracheal tube or nasogastric tube), and drain (drainage tube from body cavities or wounds)
Equipment/devices	Errors related to medical equipment and devices
Diagnostic testing	Errors related to laboratory and imaging tests
Therapeutic	Errors related to treatments or procedures
Nursing care	Errors related to nursing care
Miscellaneous	None of the above

**Table 2:** Classification of the level of severity and influence of errors recommended by the National University Hospital Council of Japan.

Level	Continuity of injury	Severity of injury	Description (NCC MERP^a^ Category)
0	None	N/A^b^	Errors or malfunctions in medicines and medical devices occurred but did not reach the patient (B).
1	None	N/A	There was no actual harm to the patient (but there was a possibility of some influence) (C).
2	Transient	Mild	Treatment was not required (enhanced patient observation, mild change in vital signs, examination for confirmation of safety, etc) (D).
3a	Transient	Moderate	A simple procedure or treatment was required (disinfection, poultice, skin suture, administration of analgesics) (E).
3b	Transient	Severe	A substantial procedure or treatment was required (significant change in vital signs, use of mechanical ventilation, surgery, prolongation of hospital stay, hospitalization, fracture, etc.) (F).
4a	Permanent	Mild-moderate	Permanent disability or sequelae remained without significant functional impairment or cosmetic problems (G or H).
4b	Permanent	Moderate-severe	Permanent disability or sequelae remained with significant functional impairment or cosmetic problems (G or H).
5	Death	N/A	Death (excluding that due to the natural course of the underlying disease) (I).
Others	N/A	N/A	Errors to which the classification was not able to be applied.

^a^NCC MERP: National Coordinating Council for Medication Error Reporting and Prevention.

^b^N/A: not applicable.

### Statistical Analysis

Categorical variables are presented as frequencies and percentages, and Fisher exact test was used to analyze statistical significance. Continuous variables are presented as median and IQR, and we used a nonparametric test (Mann-Whitney test) for continuous variables. The incidence rate of errors was calculated as the number of events per 1000 patient-days. The incidence rates of errors in the study periods were compared by their 95% CIs calculated using the Poisson distribution and exact conditional test. Statistical significance was set at *P*<.05. All statistical analyses were conducted using R Statistical Package 4.1.1 (The R Foundation for Statistical Computing).

## Results

### Demographics

From April 1, 2018, to September 30, 2018, 755 patients were admitted to the ICU, and 719 patients were admitted from April 1, 2019, to September 30, 2019. The total lengths of stay during the 2018 and 2019 study periods were 2828 and 2600 patient-days, respectively (Table 3). The median lengths of stay in 2018 and 2019 were similar (1.6 days). The compositions of the treatment departments of patients were also similar between 2018 and 2019, and cardiovascular surgery, neurosurgery, thoracic surgery, and gastrointestinal surgery were the major departments. The patient characteristics were comparable between 2018 and 2019. The ICU was staffed with a 1:2 nurse to patient ratio with 9 intensivists (3 to 4 during weekdays and 1 at night and on weekends). Staffing did not change between the 2 periods.

**Table 3:** Demographic data of the intensive care unit.

		Apr-Sep 2018	Apr-Sep 2019	*P* value	
Patients admitted, n		755	719	N/A^a^	
Total length of stay (patient-days), n		2828	2600	N/A	
Length of stay (days), median (IQR)		1.6 (0.8-3.6)	1.6 (0.9-3.0)	.24	
**Patient characteristics**					
	Age (years), median (IQR)	63 (47-74)	64 (45-72)	.42	
	Male gender, n (%)	434 (57.5)	420 (58.4)	.76	
**Race**					.93
	Asian-Japanese, n (%)	747 (98.9)	711 (98.9)		
	Asian-other, n (%)	4 (0.5)	5 (0.7)		
	White, n (%)	2 (0.3)	2 (0.3)		
	Other, n (%)	2 (0.3)	2 (0.3)		
**Patients by treatment department**					.49
	Cardiovascular surgery, n (%)	264 (35.0)	268 (37.3)		
	Neurosurgery, n (%)	242 (32.1)	196 (27.3)		
	Gastrointestinal surgery, n (%)	68 (9.0)	65 (9.0)		
	Thoracic surgery, n (%)	80 (10.6)	95 (13.0)		
	Urology and renal transplantation, n (%)	31 (4.1)	30 (4.2)		
	Endocrine surgery, n (%)	5 (0.7)	3 (0.4)		
	Miscellaneous surgery, n (%)	24 (3.2)	19 (2.6)		
	Medical, n (%)	41 (5.4)	43 (5.9)		

^a^N/A: not applicable.

### Evaluation Outcomes

The number of errors was 156 in the 2018 study period and 71 in the 2019 study period. The error incidence rates in 2018 and 2019 were 55.2 (95% CI 46.8-64.5) and 27.3 (95% CI 21.3-34.4) events per 1000 patient-days, respectively (*P*<.001; Table 4). Approximately 40% of errors occurred in patients treated in the cardiovascular surgery department in both periods, and there was no significant difference in the composition of treatment departments in which the errors occurred.

The number and incidence rate of ICIS-related errors in the 2019 study period were 10 (10/71, 14%) and 3.8 (95% CI 1.8-7.1) events per 1000 patient-days, respectively (Table 4). All ICIS-related errors were associated with the CPOE component of the ICIS, and the major background factors of the errors were inadequate coordination between the ICIS and EMR system (4 events due to an inability to synchronize orders between both systems), unfamiliarity with ICIS operations (3 events), inadequate confirmation (2 events), and specifications of the ICIS (1 event).

During both the periods, there were no significant changes in the composition of the types of errors (*P*=.14), and the most common type of error was medication error (Table 5). The number of errors related to medication decreased from 78 in 2018 (78/156, 50.0%) to 31 in 2019 (31/71, 43.7%), and the incidence rate of medication errors significantly decreased from 27.5 events per 1000 patient-days in 2018 (95% CI 21.8-34.4) to 11.9 events per 1000 patient-days in 2019 (95% CI 8.1-16.9; Table 5). The second most common type of error was errors related to line, tube, or drain. The number of errors decreased from 53 in 2018 (53/156, 34.0%) to 24 in 2019 (24/71, 33.8%), but the percentage of total errors remained the same. The incidence rate of errors related to line, tube, or drain significantly decreased from 18.7 events per 1000 patient-days in 2018 (95% CI 14.0-24.5) to 9.2 events per 1000 patient-days in 2019 (95% CI 5.9-13.7; Table 5). There was no difference in the severity and influence level of errors in both periods (*P*=.59), and level 1 and 2 errors accounted for most of the errors. The incidence rate in level 1 errors was reduced by one-third, and the incidence rate in each of the level 2 and 3a errors was reduced by one-half (Table 6).

**Table 4:** Errors in the periods of April 2018 to September 2018 and April 2019 to September 2019.

			Apr-Sep 2018	Apr-Sep 2019	*P* value	
Total errors, n			156	71	N/A^a^	
Incidence rate of total errors, events per 1000 patient-days (95% CI)			55.2 (46.8-64.5)	27.3 (21.3-34.4)	<.001	
**Errors by treatment department, n (%)**						.18
	Cardiovascular surgery	59 (37.8)		30 (42.3)		
	Neurosurgery	25 (16.0)		11 (15.5)		
	Gastrointestinal surgery	25 (16.0)		13 (18.3)		
	Thoracic surgery	5 (3.2)		0 (0.0)		
	Urology and renal transplantation	6 (3.8)		5 (7.0)		
	Endocrine surgery	0 (0.0)		0 (0.0)		
	Miscellaneous surgery	1 (0.6)		2 (2.8)		
	Medical	34 (21.8)		8 (11.3)		
	Nondepartment	1 (0.6)		2 (2.8)		
ICIS^b^-related errors, n (%)			N/A	10 (14.1)	N/A	
ICIS-related errors incidence rate, events per 1000 patient-days (95% CI)			N/A	3.8 (1.8-7.1)	N/A	

^a^N/A: not applicable.

^b^ICIS: intensive care information system.

**Table 5:** Type of errors in periods of April 2018 to September 2018 and April 2019 to September 2019.

Type of errors	Apr-Sep 2018		Apr-Sep 2019			*P* value
	n (%) (N=156)	Incidence rate^a^	n (%) (N=71)	Incidence rate^a^		
Medication	78 (50.0)	27.5 (21.8-34.4)	31 (43.7)	11.9 (8.1-16.9)	<.001	
Line, tube, or drain	53 (34.0)	18.7 (14.0-24.5)	24 (33.8)	9.2 (5.9-13.7)	.004	
Equipment/devices	11 (7.1)	3.9 (1.9-7.0)	4 (5.6)	1.5 (0.4-3.9)	.12	
Diagnostic testing	6 (3.8)	2.1 (0.8-4.6)	1 (1.4)	0.4 (0.01-2.1)	.13	
Therapeutic	1 (0.6)	0.4 (0.01-2.0)	3 (4.2)	1.2 (0.2-3.4)	.36	
Nursing care	6 (3.8)	2.1 (0.8-4.6)	5 (7.0)	1.9 (0.6-4.5)	>.99	
Miscellaneous	1 (0.6)	0.4 (0.01-2.0)	3 (4.2)	1.2 (0.2-3.4)	.36	

^a^The incidence rate of the type of errors is presented as events per 1000 patient-days and 95% CI.

**Table 6:** Severity and influence level of errors in periods of April 2018 to September 2018 and April 2019 to September 2019.

Level of errors	Apr-Sep 2018			Apr-Sep 2019		*P* value
	n (%) (N=156)	Incidence rate^a^	n (%) (N = 71)		Incidence rate^a^	
Level 0	23 (14.7)	8.1 (5.2-12.2)	7 (9.9)		2.7 (1.1-5.5)	.009
Level 1	44 (28.2)	15.2 (11.3-20.9)	26 (36.6)		10.0 (6.5-14.7)	.07
Level 2	47 (30.1)	16.6 (12.2-22.1)	19 (26.8)		7.3 (4.4-11.4)	.002
Level 3a	33 (21.2)	11.7 (8.0-16.4)	14 (19.7)		5.4 (2.9-9.0)	.01
Level 3b	7 (4.5)	2.5 (1.0-5.1)	5 (7.0)		1.9 (0.6-4.5)	.78
Level 4a	0 (0.0)	0	0 (0.0)		0	N/A^b^
Level 4b	0 (0.0)	0	0 (0.0)		0	N/A
Level 5	0 (0.0)	0	0 (0.0)		0	N/A
Others	2 (1.3)	0.7 (0.09-2.6)	0 (0.0)		0	N/A

^a^The incidence rate of the level of errors is presented as events per 1000 patient-days and 95% CI.

^b^N/A: not applicable.

## Discussion

### Principal Results

Three important clinical observations were made in this study. First, the number and incidence rate of errors after ICIS implementation in the ICU were halved compared with those before the implementation. Second, the most common type of error was medication error before and after implementation, and the number and incidence rate of errors related to medication significantly decreased. Third, 14% (10/71) of the errors after the implementation were relevant to the ICIS.

The incidence of errors in the ICU differs between a study and its settings. In a study on the nature and incidence of adverse events and medical errors, the incidence rate of adverse events in the medical ICU and coronary care unit was 80.5 events per 1000 patient-days [18]. The incidence rate of critical incidents in a multidisciplinary ICU was 34 events per 1000 patient-days [19]. In the study of a voluntary card-based event reporting system in 3 ICUs, the incidence rates of reported patient safety events were 55.5, 25.3, and 40.3 events per 1000 patient-days in the medical ICU, cardiothoracic ICU, and surgical ICU, respectively [20]. In addition, the incidence rate of patient safety events differed by ICU intensity: 44.1 and 24.9 events per 1000 patient-days in level 3 (higher intensity) and level 2 (lower intensity) ICUs, respectively [21]. Considering the severity and influence level of errors reported in this study, the error incidence rates for both periods were comparable to those reported in previous studies.

Although the various benefits of ICIS implementation have been reported, the role of an ICIS in preventing errors in an ICU has not been clarified. Several studies reported a decrease in documentation and charting time after ICIS implementation, thus leaving more time for patient assessment, patient care, and other nursing activities [2,4,22,23]. A study on the relationship between patient safety and nursing workloads showed that higher nursing workloads might be related to a greater number of patient safety incidents in general wards [24]; that is, the workload of ICU nurses can affect the incidence of errors. Considering that the number of patients, total length of stay, and length of ICU stay were similar for both study periods, the changes in nurses’ workload and increased productivity from the workload reduction by ICIS might have contributed to error reduction.

Furthermore, the simplification and integration of drug prescription and the presentation of information by ICIS might have contributed to an improvement in the quality of patient care by the ICU staff. Considering that the CPOE and flowsheet of the EMR system used for ICU patients before ICIS implementation were not optimized for critical care settings, paper-based orders were used simultaneously. A study on the effect of EMR implementation on medical ICUs reported that the incidence rate of medication errors increased after the implementation despite the survival benefits [25]. The composition of the user interface within the ICU electronic environment has been reported to affect the task load, task completion time, number of cognition errors related to identification, and subsequent use of patient data [7]. In addition, the use of dashboards that visualize electronic health record information has been reported to decrease the time and difficulty of data gathering; reduce cognitive load, time to task completion, and errors; and improve situation awareness [26]. Improvements in the user interface with ICIS might have led to a reduction in both workload and errors.

As discussed, medication is a major cause of errors in the ICU, and incidence rates of errors related to medication have been reported to range from 1.2 to 947 errors per 1000 patient-days [18,27-30]. The incidence rate has been reported to be higher in medical ICUs than in surgical ICUs [31]. The administration of parenteral drugs, including catecholamines and vasopressors, analgesics and sedatives, antimicrobials, coagulation-related drugs, insulin, and electrolytes, have been found to be associated with errors in the ICU [29]. Frequent dosage changes of these drugs, such as after cardiac surgery, can also increase the risk of medication errors.

The number and incidence rate of medication errors significantly decreased after ICIS implementation in this study. The influence of the implementation of an integrated ICIS on the incidence of medication errors in ICUs is not well documented. A study comparing a paper-based ICU and a computerized ICU 10 months after ICIS implementation reported significantly lower incidence and severity of medication prescription errors in an ICU using an ICIS than in a paper-based ICU [11]. By contrast, several studies have addressed the effect of CPOE implementation on the incidence of medication errors. Some studies reported that CPOE implementation in the ICU significantly reduced the incidence of medication errors compared to paper-based orders, whereas another study reported that duplicate orders of medication increased after CPOE and CDSS implementation [12,13,32-35]. The guidelines for safe medication use in the ICU recommend CPOE implementation to decrease medication errors and prevent adverse drug events [36]. Given that CPOE is a major component of an ICIS, the reduction in the number and incidence rate of medication errors in this study could be attributed to the implementation of the ICIS.

In this study, the number and incidence rate of errors related to line, tube, or drain also significantly decreased after ICIS implementation. Mion et al [37] reported that the incidence rate of patient-initiated device removal was 22.1 events per 1000 patient days. In another study, the incidence rate of the accidental removal of devices was 2.3 events per 1000 device-days, and the most frequently removed device was a gastric tube (10.2 events per 1000 device-days) [38]. Unlike medication errors, the ICIS did not have a function that was directly related to the reduction of errors related to line, tube, or drain. However, the changes in nurses’ workload by the ICIS might have contributed to the error reduction.

In this study, 14% (10/71) of the errors after ICIS implementation were relevant to the ICIS. Although the incidence of errors related to an integrated ICIS with several components is not well documented, the results of studies on CPOE may be applied since it contributed to ICIS-related errors in this study. In a study on duplicate medication order errors, 13% of incidents in medical ICUs and 6% in surgical ICUs were reported to be CPOE related, and the incidence rate of duplicate orders of medication increased from 11.6 errors per 1000 patient-days to 41.6 errors per 1000 patient-days after CPOE implementation [35,39]. These percentages and incidence rates of errors are comparable to the results of our study.

### Limitations

This study has several limitations. First, this study was performed in a single institution with a single ICIS and with a single combination of an ICIS and EMR system. Given that the work environment and human resources in an ICU vary from hospital to hospital, the type, number, and incidence rate of errors can be affected by differences in facilities. Furthermore, there are many systems in ICISs and EMRs, and their combinations have many patterns. Therefore, the settings in which the system is used also differ between an ICU and a hospital. However, no research has examined the changes in medical errors before and after ICIS implementation in an ICU where an EMR system is already in use, and we are convinced that this is one of the strengths of this study. Second, owing to the before-and-after design nature of this study, bias in both the 2018 and 2019 study periods cannot be excluded. However, given that there was no significant difference in the number of patients, patient days, the length of ICU stay, or the composition of treatment departments of patients in the ICU during the 2 periods, we believe that the situation surrounding the ICU staff has not changed remarkably. In addition, most medical staff continued to perform the same ICU duties during both periods. Third, the voluntary self-reporting system has limitations in that the reporting of errors depends on the ICU staff and on the culture and atmosphere for reporting errors in departments or organizations; thus, all errors may not be completely reported. As a result, underreporting of small errors may occur, leading to some bias. However, given that the composition of the level of errors was similar in both periods and that the ICU staff were regularly educated about medical safety, their attitudes toward error reporting and the culture and atmosphere of the ICU toward errors did not change significantly.

### Conclusions

We performed a retrospective analysis of the errors in the ICU before and after ICIS implementation in a setting where an EMR system is already in use. ICIS implementation was temporally associated with a 50% reduction in the number and incidence rate of errors in the ICU. Although the most common type of error was medication error in both study periods, the number and incidence rate of medication errors significantly decreased after ICIS implementation. The ICIS-related errors accounted for 14% (10/71) of the errors after the implementation. Our analysis suggests that ICIS could play a pivotal role in preventing errors even in a setting where an EMR system is already in use.

## Abbreviations

CDSS: clinical decision support system
CPOE: computerized physician order entry
EMR: electronic medical record
ICIS: intensive care information system
ICU: intensive care unit

## Data Availability

The data sets generated during or analyzed during this study are available from the corresponding author on reasonable request.
